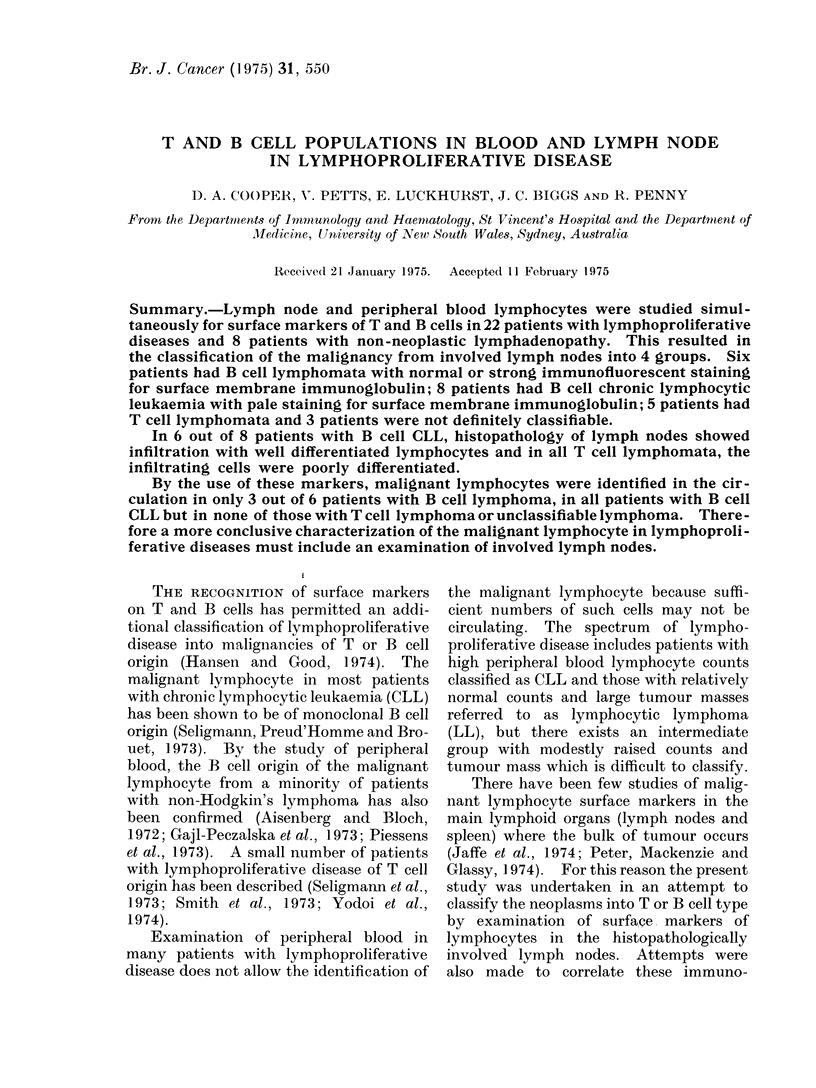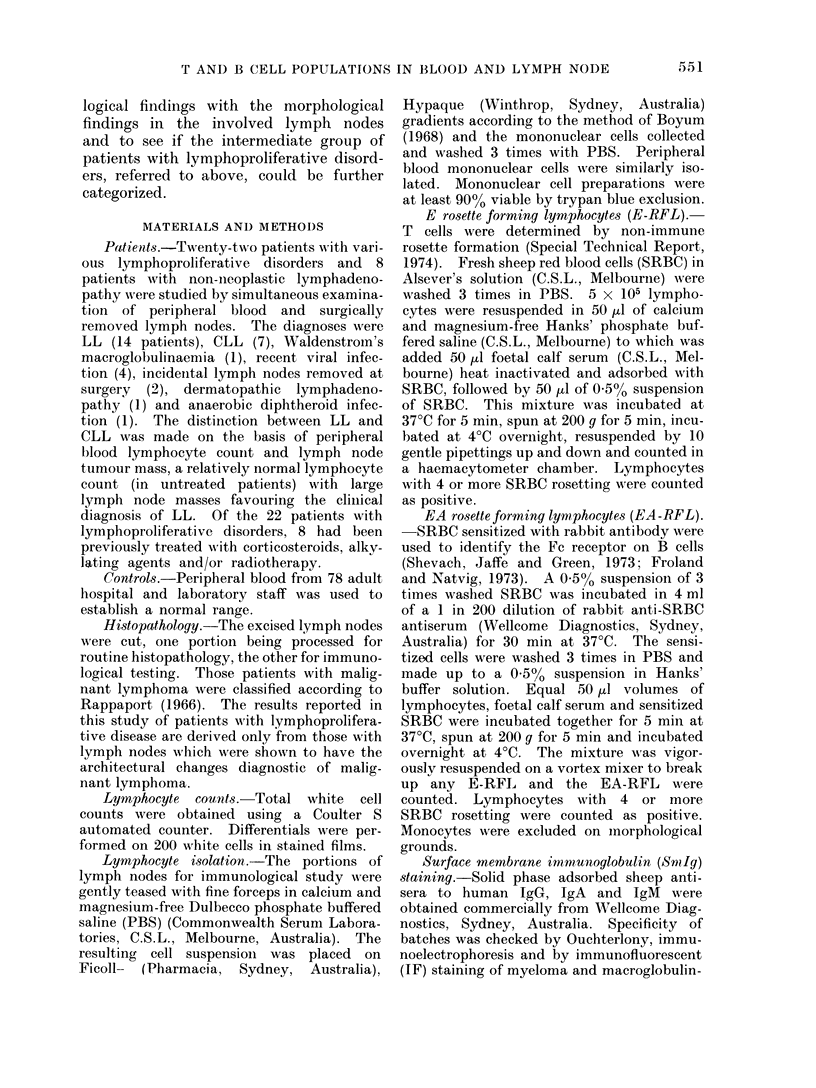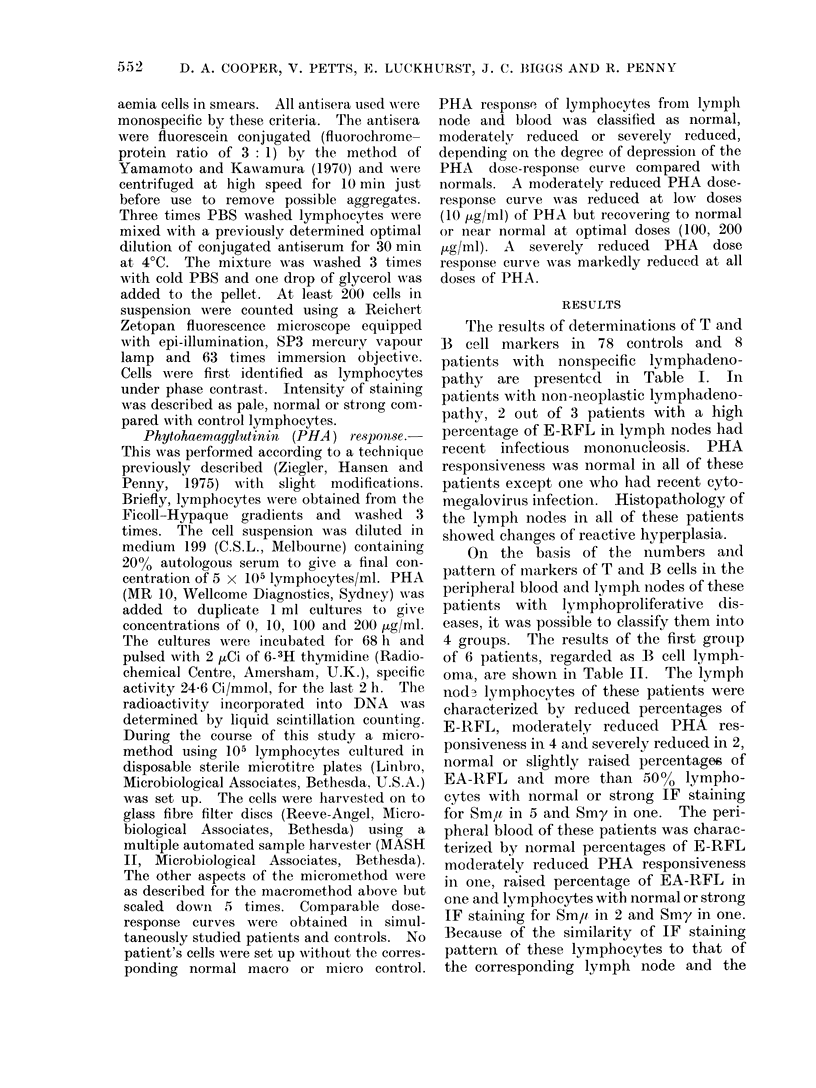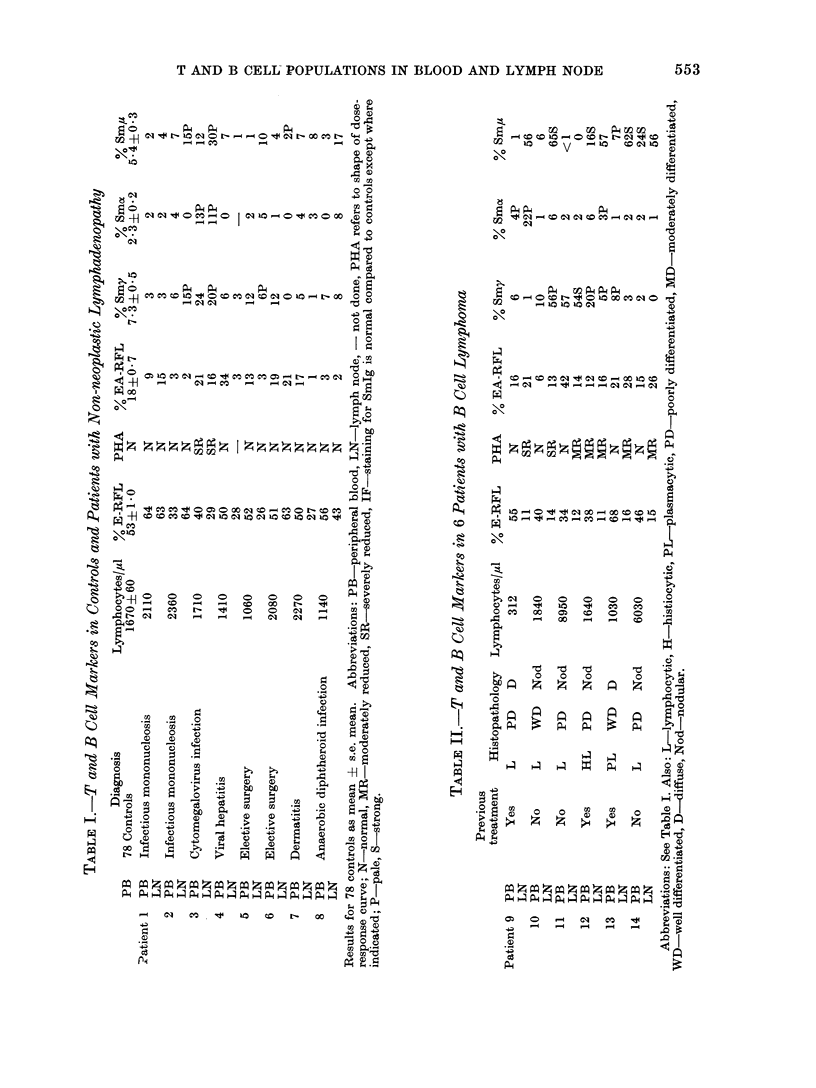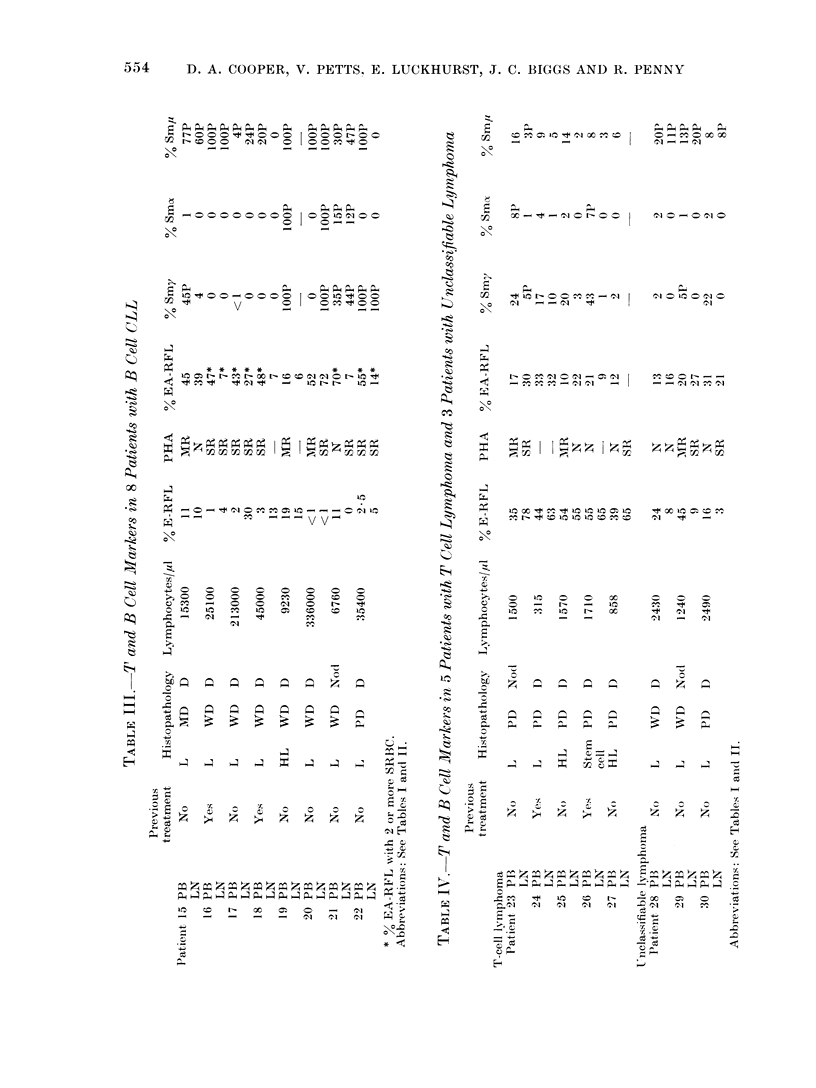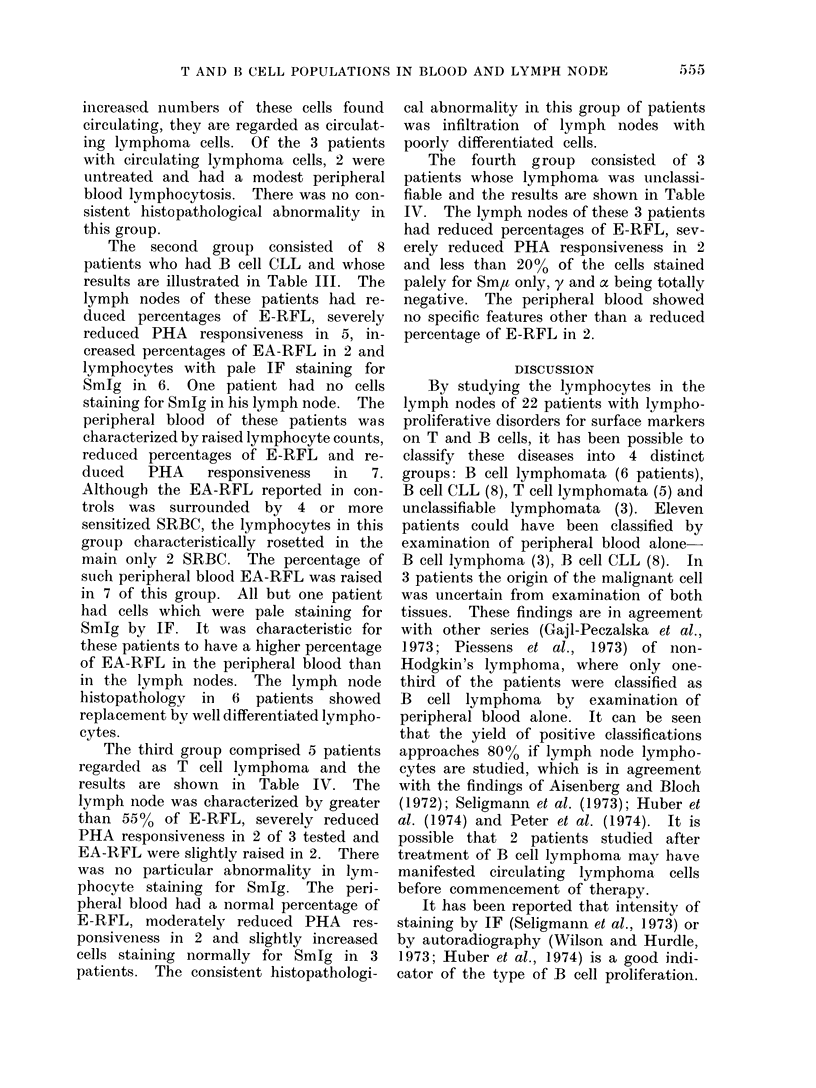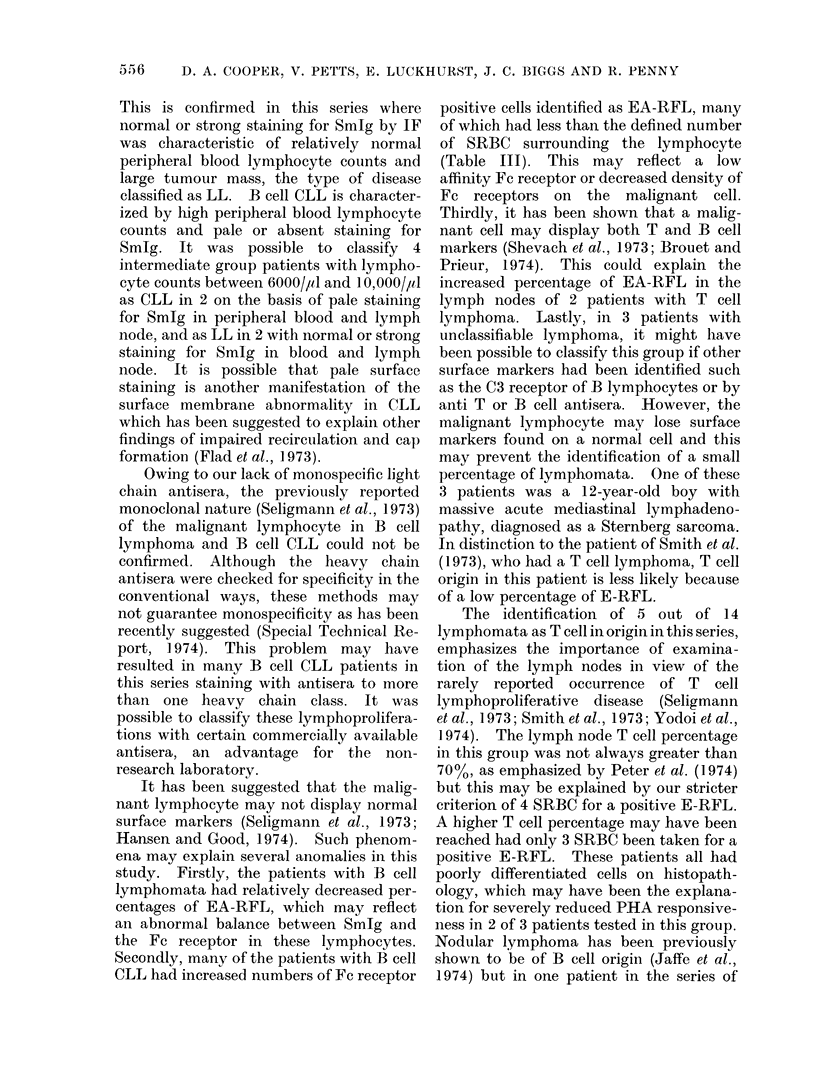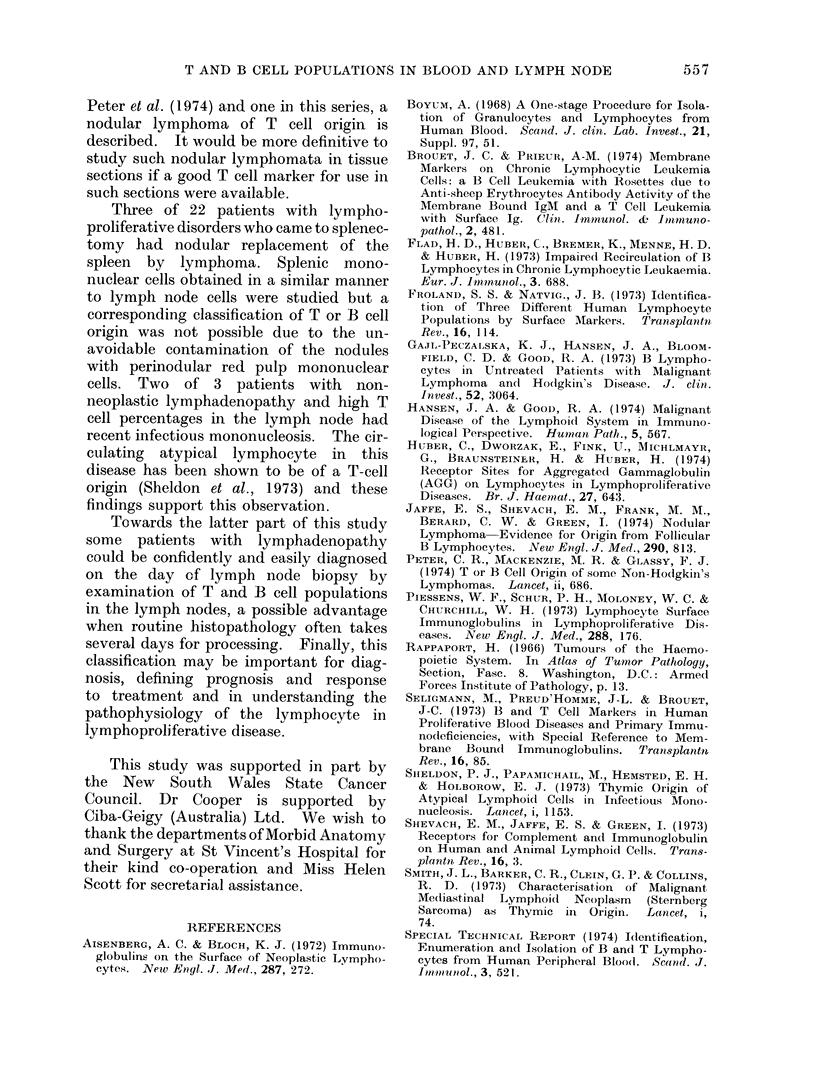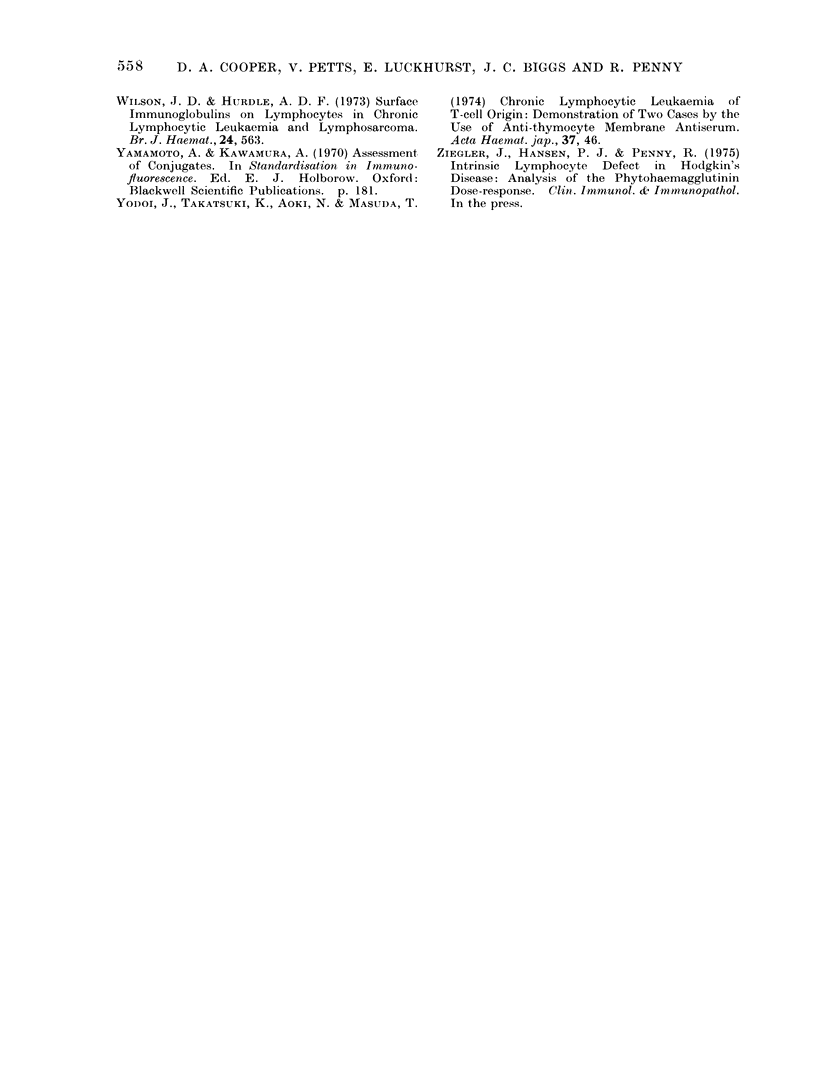# T and B cell populations in blood and lymph node in lymphoproliferative disease.

**DOI:** 10.1038/bjc.1975.96

**Published:** 1975-05

**Authors:** D. A. Cooper, V. Petts, E. Luckhurst, J. C. Biggs, R. Penny

## Abstract

Lymph node and peripheral blood lymphocytes were studied simultaneously for surface markers of T and B cells in 22 patients with lymphoproliferative diseases and 8 patients with non-neoplastic lymphadenopathy. This resulted in the classification of the malignancy from involved lymph nodes into 4 groups. Six patients had B cell lymphomata with normal or strong immunofluorescent staining for surface membrane immunoglobulin; 8 patients had B cell chronic lymphocytic leukaemia with pale staining for surface membrane immunoglobulin; 5 patients had T cell lymphomata and 3 patients were not definitely classifiable. In 6 out of 8 patients with B cell CLL, histopathology of lymph nodes showed infiltration with well differentiated lymphocytes and in all T cell lymphomata, the infiltrating cells were poorly differentiated. By the use of these markers, malignant lymphocytes were identified in the circulation in only 3 out of 6 patients with B cell lymphoma, in all patients with B cell CLL but in none of those with T cell lymphoma or unclassifiable lymphoma. Therefore a more conclusive characterization of the malignant lymphocyte in lymphoproliferative diseases must include an examination of involved lymph nodes.


*Br. J. Cancer* (1975) **31**, 550

### T AND B CELL POPULATIONS IN BLOOD AND LYMPH NODE IN LYMPHOPROLIFERATIVE DISEASE

D. A. COOPER, V. PETTS, E. LUCKHURST, J. C. BIGGS AND R. PENNY

*From the Departments of Immunology and Haematology, St Vincent's Hospital and the Department of Medicine, University of New South Wales, Sydney, Australia*

Received 21 January 1975. Accepted 11 February 1975

**Summary.—Lymph node and peripheral blood lymphocytes were studied simultaneously for surface markers of T and B cells in 22 patients with lymphoproliferative diseases and 8 patients with non-neoplastic lymphadenopathy. This resulted in the classification of the malignancy from involved lymph nodes into 4 groups. Six patients had B cell lymphomata with normal or strong immunofluorescent staining for surface membrane immunoglobulin; 8 patients had B cell chronic lymphocytic leukaemia with pale staining for surface membrane immunoglobulin; 5 patients had T cell lymphomata and 3 patients were not definitely classifiable.**

**In 6 out of 8 patients with B cell CLL, histopathology of lymph nodes showed infiltration with well differentiated lymphocytes and in all T cell lymphomata, the infiltrating cells were poorly differentiated.**

**By the use of these markers, malignant lymphocytes were identified in the circulation in only 3 out of 6 patients with B cell lymphoma, in all patients with B cell CLL but in none of those with T cell lymphoma or unclassifiable lymphoma. Therefore a more conclusive characterization of the malignant lymphocyte in lymphoproliferative diseases must include an examination of involved lymph nodes.**

THE RECOGNITION of surface markers on T and B cells has permitted an additional classification of lymphoproliferative disease into malignancies of T or B cell origin (Hansen and Good, 1974). The malignant lymphocyte in most patients with chronic lymphocytic leukaemia (CLL) has been shown to be of monoclonal B cell origin (Seligmann, Preud'Homme and Brouet, 1973). By the study of peripheral blood, the B cell origin of the malignant lymphocyte from a minority of patients with non-Hodgkin's lymphoma has also been confirmed (Aisenberg and Bloch, 1972; Gajl-Peczalska *et al.*, 1973; Piessens *et al.*, 1973). A small number of patients with lymphoproliferative disease of T cell origin has been described (Seligmann *et al.*, 1973; Smith *et al.*, 1973; Yodoi *et al.*, 1974).

Examination of peripheral blood in many patients with lymphoproliferative disease does not allow the identification of the malignant lymphocyte because sufficient numbers of such cells may not be circulating. The spectrum of lymphoproliferative disease includes patients with high peripheral blood lymphocyte counts classified as CLL and those with relatively normal counts and large tumour masses referred to as lymphocytic lymphoma (LL), but there exists an intermediate group with modestly raised counts and tumour mass which is difficult to classify.

There have been few studies of malignant lymphocyte surface markers in the main lymphoid organs (lymph nodes and spleen) where the bulk of tumour occurs (Jaffe *et al.*, 1974; Peter, Mackenzie and Glassy, 1974). For this reason the present study was undertaken in an attempt to classify the neoplasms into T or B cell type by examination of surface markers of lymphocytes in the histopathologically involved lymph nodes. Attempts were also made to correlate these immunological findings with the morphological findings in the involved lymph nodes and to see if the intermediate group of patients with lymphoproliferative disorders, referred to above, could be further categorized.

### MATERIALS AND METHODS

*Patients*.—Twenty-two patients with various lymphoproliferative disorders and 8 patients with non-neoplastic lymphadenopathy were studied by simultaneous examination of peripheral blood and surgically removed lymph nodes. The diagnoses were LL (14 patients), CLL (7), Waldenstrom's macroglobulinaemia (1), recent viral infection (4), incidental lymph nodes removed at surgery (2), dermatopathic lymphadenopathy (1) and anaerobic diphtheroid infection (1). The distinction between LL and CLL was made on the basis of peripheral blood lymphocyte count and lymph node tumour mass, a relatively normal lymphocyte count (in untreated patients) with large lymph node masses favouring the clinical diagnosis of LL. Of the 22 patients with lymphoproliferative disorders, 8 had been previously treated with corticosteroids, alkylating agents and/or radiotherapy.

*Controls*.—Peripheral blood from 78 adult hospital and laboratory staff was used to establish a normal range.

*Histopathology*.—The excised lymph nodes were cut, one portion being processed for routine histopathology, the other for immunological testing. Those patients with malignant lymphoma were classified according to Rappaport (1966). The results reported in this study of patients with lymphoproliferative disease are derived only from those with lymph nodes which were shown to have the architectural changes diagnostic of malignant lymphoma.

*Lymphocyte counts*.—Total white cell counts were obtained using a Coulter S automated counter. Differentials were performed on 200 white cells in stained films.

*Lymphocyte isolation*.—The portions of lymph nodes for immunological study were gently teased with fine forceps in calcium and magnesium-free Dulbecco phosphate buffered saline (PBS) (Commonwealth Serum Laboratories, C.S.L., Melbourne, Australia). The resulting cell suspension was placed on Ficoll- (Pharmacia, Sydney, Australia), Hypaque (Winthrop, Sydney, Australia) gradients according to the method of Boyum (1968) and the mononuclear cells collected and washed 3 times with PBS. Peripheral blood mononuclear cells were similarly isolated. Mononuclear cell preparations were at least 90% viable by trypan blue exclusion.

*E rosette forming lymphocytes* (*E-RFL*).—T cells were determined by non-immune rosette formation (Special Technical Report, 1974). Fresh sheep red blood cells (SRBC) in Alsever's solution (C.S.L., Melbourne) were washed 3 times in PBS. $5 \times 10^5$ lymphocytes were resuspended in 50 $\mu$l of calcium and magnesium-free Hanks' phosphate buffered saline (C.S.L., Melbourne) to which was added 50 $\mu$l foetal calf serum (C.S.L., Melbourne) heat inactivated and adsorbed with SRBC, followed by 50 $\mu$l of 0·5% suspension of SRBC. This mixture was incubated at 37°C for 5 min, spun at 200 *g* for 5 min, incubated at 4°C overnight, resuspended by 10 gentle pipettings up and down and counted in a haemacytometer chamber. Lymphocytes with 4 or more SRBC rosetting were counted as positive.

*EA rosette forming lymphocytes* (*EA-RFL*).—SRBC sensitized with rabbit antibody were used to identify the Fc receptor on B cells (Shevach, Jaffe and Green, 1973; Froland and Natvig, 1973). A 0·5% suspension of 3 times washed SRBC was incubated in 4 ml of a 1 in 200 dilution of rabbit anti-SRBC antiserum (Wellcome Diagnostics, Sydney, Australia) for 30 min at 37°C. The sensitized cells were washed 3 times in PBS and made up to a 0·5% suspension in Hanks' buffer solution. Equal 50 $\mu$l volumes of lymphocytes, foetal calf serum and sensitized SRBC were incubated together for 5 min at 37°C, spun at 200 *g* for 5 min and incubated overnight at 4°C. The mixture was vigorously resuspended on a vortex mixer to break up any E-RFL and the EA-RFL were counted. Lymphocytes with 4 or more SRBC rosetting were counted as positive. Monocytes were excluded on morphological grounds.

*Surface membrane immunoglobulin* (*SmIg*) *staining*.—Solid phase adsorbed sheep antisera to human IgG, IgA and IgM were obtained commercially from Wellcome Diagnostics, Sydney, Australia. Specificity of batches was checked by Ouchterlony, immunoelectrophoresis and by immunofluorescent (IF) staining of myeloma and macroglobulinaemia cells in smears. All antisera used were monospecific by these criteria. The antisera were fluorescein conjugated (fluorochrome–protein ratio of 3 : 1) by the method of Yamamoto and Kawamura (1970) and were centrifuged at high speed for 10 min just before use to remove possible aggregates. Three times PBS washed lymphocytes were mixed with a previously determined optimal dilution of conjugated antiserum for 30 min at 4°C. The mixture was washed 3 times with cold PBS and one drop of glycerol was added to the pellet. At least 200 cells in suspension were counted using a Reichert Zetopan fluorescence microscope equipped with epi-illumination, SP3 mercury vapour lamp and 63 times immersion objective. Cells were first identified as lymphocytes under phase contrast. Intensity of staining was described as pale, normal or strong compared with control lymphocytes.

*Phytohaemagglutinin* (*PHA*) *response.*—This was performed according to a technique previously described (Ziegler, Hansen and Penny, 1975) with slight modifications. Briefly, lymphocytes were obtained from the Ficoll–Hypaque gradients and washed 3 times. The cell suspension was diluted in medium 199 (C.S.L., Melbourne) containing 20% autologous serum to give a final concentration of $5 \times 10^5$ lymphocytes/ml. PHA (MR 10, Wellcome Diagnostics, Sydney) was added to duplicate 1 ml cultures to give concentrations of 0, 10, 100 and 200 $\mu$g/ml. The cultures were incubated for 68 h and pulsed with 2 $\mu$Ci of 6-$^3$H thymidine (Radiochemical Centre, Amersham, U.K.), specific activity 24·6 Ci/mmol, for the last 2 h. The radioactivity incorporated into DNA was determined by liquid scintillation counting. During the course of this study a micromethod using $10^5$ lymphocytes cultured in disposable sterile microtitre plates (Linbro, Microbiological Associates, Bethesda, U.S.A.) was set up. The cells were harvested on to glass fibre filter discs (Reeve-Angel, Microbiological Associates, Bethesda) using a multiple automated sample harvester (MASH II, Microbiological Associates, Bethesda). The other aspects of the micromethod were as described for the macromethod above but scaled down 5 times. Comparable dose-response curves were obtained in simultaneously studied patients and controls. No patient's cells were set up without the corresponding normal macro or micro control. PHA response of lymphocytes from lymph node and blood was classified as normal, moderately reduced or severely reduced, depending on the degree of depression of the PHA dose-response curve compared with normals. A moderately reduced PHA dose-response curve was reduced at low doses (10 $\mu$g/ml) of PHA but recovering to normal or near normal at optimal doses (100, 200 $\mu$g/ml). A severely reduced PHA dose response curve was markedly reduced at all doses of PHA.

### RESULTS

The results of determinations of T and B cell markers in 78 controls and 8 patients with nonspecific lymphadenopathy are presented in Table I. In patients with non-neoplastic lymphadenopathy, 2 out of 3 patients with a high percentage of E-RFL in lymph nodes had recent infectious mononucleosis. PHA responsiveness was normal in all of these patients except one who had recent cytomegalovirus infection. Histopathology of the lymph nodes in all of these patients showed changes of reactive hyperplasia.

On the basis of the numbers and pattern of markers of T and B cells in the peripheral blood and lymph nodes of these patients with lymphoproliferative diseases, it was possible to classify them into 4 groups. The results of the first group of 6 patients, regarded as B cell lymphoma, are shown in Table II. The lymph node lymphocytes of these patients were characterized by reduced percentages of E-RFL, moderately reduced PHA responsiveness in 4 and severely reduced in 2, normal or slightly raised percentages of EA-RFL and more than 50% lymphocytes with normal or strong IF staining for Sm$\mu$ in 5 and Sm$\gamma$ in one. The peripheral blood of these patients was characterized by normal percentages of E-RFL moderately reduced PHA responsiveness in one, raised percentage of EA-RFL in one and lymphocytes with normal or strong IF staining for Sm$\mu$ in 2 and Sm$\gamma$ in one. Because of the similarity of IF staining pattern of these lymphocytes to that of the corresponding lymph node and the increased numbers of these cells found circulating, they are regarded as circulating lymphoma cells. Of the 3 patients with circulating lymphoma cells, 2 were untreated and had a modest peripheral blood lymphocytosis. There was no consistent histopathological abnormality in this group.

The second group consisted of 8 patients who had B cell CLL and whose results are illustrated in Table III. The lymph nodes of these patients had reduced percentages of E-RFL, severely reduced PHA responsiveness in 5, increased percentages of EA-RFL in 2 and lymphocytes with pale IF staining for SmIg in 6. One patient had no cells staining for SmIg in his lymph node. The peripheral blood of these patients was characterized by raised lymphocyte counts, reduced percentages of E-RFL and reduced PHA responsiveness in 7. Although the EA-RFL reported in controls was surrounded by 4 or more sensitized SRBC, the lymphocytes in this group characteristically rosetted in the main only 2 SRBC. The percentage of such peripheral blood EA-RFL was raised in 7 of this group. All but one patient had cells which were pale staining for SmIg by IF. It was characteristic for these patients to have a higher percentage of EA-RFL in the peripheral blood than in the lymph nodes. The lymph node histopathology in 6 patients showed replacement by well differentiated lymphocytes.

The third group comprised 5 patients regarded as T cell lymphoma and the results are shown in Table IV. The lymph node was characterized by greater than 55% of E-RFL, severely reduced PHA responsiveness in 2 of 3 tested and EA-RFL were slightly raised in 2. There was no particular abnormality in lymphocyte staining for SmIg. The peripheral blood had a normal percentage of E-RFL, moderately reduced PHA responsiveness in 2 and slightly increased cells staining normally for SmIg in 3 patients. The consistent histopathological abnormality in this group of patients was infiltration of lymph nodes with poorly differentiated cells.

The fourth group consisted of 3 patients whose lymphoma was unclassifiable and the results are shown in Table IV. The lymph nodes of these 3 patients had reduced percentages of E-RFL, severely reduced PHA responsiveness in 2 and less than 20% of the cells stained palely for Sm$\mu$ only, $\gamma$ and $\alpha$ being totally negative. The peripheral blood showed no specific features other than a reduced percentage of E-RFL in 2.

### DISCUSSION

By studying the lymphocytes in the lymph nodes of 22 patients with lymphoproliferative disorders for surface markers on T and B cells, it has been possible to classify these diseases into 4 distinct groups: B cell lymphomata (6 patients), B cell CLL (8), T cell lymphomata (5) and unclassifiable lymphomata (3). Eleven patients could have been classified by examination of peripheral blood alone—B cell lymphoma (3), B cell CLL (8). In 3 patients the origin of the malignant cell was uncertain from examination of both tissues. These findings are in agreement with other series (Gajl-Peczalska *et al.*, 1973; Piessens *et al.*, 1973) of non-Hodgkin's lymphoma, where only one-third of the patients were classified as B cell lymphoma by examination of peripheral blood alone. It can be seen that the yield of positive classifications approaches 80% if lymph node lymphocytes are studied, which is in agreement with the findings of Aisenberg and Bloch (1972); Seligmann *et al.* (1973); Huber *et al.* (1974) and Peter *et al.* (1974). It is possible that 2 patients studied after treatment of B cell lymphoma may have manifested circulating lymphoma cells before commencement of therapy.

It has been reported that intensity of staining by IF (Seligmann *et al.*, 1973) or by autoradiography (Wilson and Hurdle, 1973; Huber *et al.*, 1974) is a good indicator of the type of B cell proliferation.

This is confirmed in this series where normal or strong staining for SmIg by IF was characteristic of relatively normal peripheral blood lymphocyte counts and large tumour mass, the type of disease classified as LL. B cell CLL is characterized by high peripheral blood lymphocyte counts and pale or absent staining for SmIg. It was possible to classify 4 intermediate group patients with lymphocyte counts between 6000/$\mu$l and 10,000/$\mu$l as CLL in 2 on the basis of pale staining for SmIg in peripheral blood and lymph node, and as LL in 2 with normal or strong staining for SmIg in blood and lymph node. It is possible that pale surface staining is another manifestation of the surface membrane abnormality in CLL which has been suggested to explain other findings of impaired recirculation and cap formation (Flad *et al.*, 1973).

Owing to our lack of monospecific light chain antisera, the previously reported monoclonal nature (Seligmann *et al.*, 1973) of the malignant lymphocyte in B cell lymphoma and B cell CLL could not be confirmed. Although the heavy chain antisera were checked for specificity in the conventional ways, these methods may not guarantee monospecificity as has been recently suggested (Special Technical Report, 1974). This problem may have resulted in many B cell CLL patients in this series staining with antisera to more than one heavy chain class. It was possible to classify these lymphoproliferations with certain commercially available antisera, an advantage for the non-research laboratory.

It has been suggested that the malignant lymphocyte may not display normal surface markers (Seligmann *et al.*, 1973; Hansen and Good, 1974). Such phenomena may explain several anomalies in this study. Firstly, the patients with B cell lymphomata had relatively decreased percentages of EA-RFL, which may reflect an abnormal balance between SmIg and the Fc receptor in these lymphocytes. Secondly, many of the patients with B cell CLL had increased numbers of Fc receptor positive cells identified as EA-RFL, many of which had less than the defined number of SRBC surrounding the lymphocyte (Table III). This may reflect a low affinity Fc receptor or decreased density of Fc receptors on the malignant cell. Thirdly, it has been shown that a malignant cell may display both T and B cell markers (Shevach *et al.*, 1973; Brouet and Prieur, 1974). This could explain the increased percentage of EA-RFL in the lymph nodes of 2 patients with T cell lymphoma. Lastly, in 3 patients with unclassifiable lymphoma, it might have been possible to classify this group if other surface markers had been identified such as the C3 receptor of B lymphocytes or by anti T or B cell antisera. However, the malignant lymphocyte may lose surface markers found on a normal cell and this may prevent the identification of a small percentage of lymphomata. One of these 3 patients was a 12-year-old boy with massive acute mediastinal lymphadenopathy, diagnosed as a Sternberg sarcoma. In distinction to the patient of Smith *et al.* (1973), who had a T cell lymphoma, T cell origin in this patient is less likely because of a low percentage of E-RFL.

The identification of 5 out of 14 lymphomata as T cell in origin in this series, emphasizes the importance of examination of the lymph nodes in view of the rarely reported occurrence of T cell lymphoproliferative disease (Seligmann *et al.*, 1973; Smith *et al.*, 1973; Yodoi *et al.*, 1974). The lymph node T cell percentage in this group was not always greater than 70%, as emphasized by Peter *et al.* (1974) but this may be explained by our stricter criterion of 4 SRBC for a positive E-RFL. A higher T cell percentage may have been reached had only 3 SRBC been taken for a positive E-RFL. These patients all had poorly differentiated cells on histopathology, which may have been the explanation for severely reduced PHA responsiveness in 2 of 3 patients tested in this group. Nodular lymphoma has been previously shown to be of B cell origin (Jaffe *et al.*, 1974) but in one patient in the series of

Peter *et al.* (1974) and one in this series, a nodular lymphoma of T cell origin is described. It would be more definitive to study such nodular lymphomata in tissue sections if a good T cell marker for use in such sections were available.

Three of 22 patients with lymphoproliferative disorders who came to splenectomy had nodular replacement of the spleen by lymphoma. Splenic mononuclear cells obtained in a similar manner to lymph node cells were studied but a corresponding classification of T or B cell origin was not possible due to the unavoidable contamination of the nodules with perinodular red pulp mononuclear cells. Two of 3 patients with non-neoplastic lymphadenopathy and high T cell percentages in the lymph node had recent infectious mononucleosis. The circulating atypical lymphocyte in this disease has been shown to be of a T-cell origin (Sheldon *et al.*, 1973) and these findings support this observation.

Towards the latter part of this study some patients with lymphadenopathy could be confidently and easily diagnosed on the day of lymph node biopsy by examination of T and B cell populations in the lymph nodes, a possible advantage when routine histopathology often takes several days for processing. Finally, this classification may be important for diagnosis, defining prognosis and response to treatment and in understanding the pathophysiology of the lymphocyte in lymphoproliferative disease.

This study was supported in part by the New South Wales State Cancer Council. Dr Cooper is supported by Ciba-Geigy (Australia) Ltd. We wish to thank the departments of Morbid Anatomy and Surgery at St Vincent's Hospital for their kind co-operation and Miss Helen Scott for secretarial assistance.

### REFERENCES

AISENBERG, A. C. & BLOCH, K. J. (1972) Immunoglobulins on the Surface of Neoplastic Lymphocytes. *New Engl. J. Med.*, **287**, 272.

BOYUM, A. (1968) A One-stage Procedure for Isolation of Granulocytes and Lymphocytes from Human Blood. *Scand. J. clin. Lab. Invest.*, **21**, Suppl. 97, 51.

BROUET, J. C. & PRIEUR, A-M. (1974) Membrane Markers on Chronic Lymphocytic Leukemia Cells: a B Cell Leukemia with Rosettes due to Anti-sheep Erythrocytes Antibody Activity of the Membrane Bound IgM and a T Cell Leukemia with Surface Ig. *Clin. Immunol. & Immunopathol.*, **2**, 481.

FLAD, H. D., HUBER, C., BREMER, K., MENNE, H. D. & HUBER, H. (1973) Impaired Recirculation of B Lymphocytes in Chronic Lymphocytic Leukaemia. *Eur. J. Immunol.*, **3**. 688.

FROLAND, S. S. & NATVIG., J. B. (1973) Identification of Three Different Human Lymphocyte Populations by Surface Markers. *Transplantn Rev.*, **16**, 114.

GAJL-PECZALSKA, K. J., HANSEN, J. A., BLOOMFIELD, C. D. & GOOD, R. A. (1973) B Lymphocytes in Untreated Patients with Malignant Lymphoma and Hodgkin's Disease. *J. clin. Invest.*, **52**, 3064.

HANSEN, J. A. & GOOD, R. A. (1974) Malignant Disease of the Lymphoid System in Immunological Perspective. *Human Path.*, **5**, 567.

HUBER, C., DWORZAK, E., FINK, U., MICHLMAYR, G., BRAUNSTEINER, H. & HUBER, H. (1974) Receptor Sites for Aggregated Gammaglobulin (AGG) on Lymphocytes in Lymphoproliferative Diseases. *Br. J. Haemat.*, **27**, 643.

JAFFE, E. S., SHEVACH, E. M., FRANK, M. M., BERARD, C. W. & GREEN, I. (1974) Nodular Lymphoma—Evidence for Origin from Follicular B Lymphocytes. *New Engl. J. Med.*, **290**, 813.

PETER, C. R., MACKENZIE, M. R. & GLASSY, F. J. (1974) T or B Cell Origin of some Non-Hodgkin's Lymphomas. *Lancet*, ii, 686.

PIESSENS, W. F., SCHUR, P. H., MOLONEY, W. C. & CHURCHILL, W. H. (1973) Lymphocyte Surface Immunoglobulins in Lymphoproliferative Diseases. *New Engl. J. Med.*, **288**, 176.

RAPPAPORT, H. (1966) Tumours of the Haemopoietic System. In *Atlas of Tumor Pathology*, Section, Fasc. 8. Washington, D.C.: Armed Forces Institute of Pathology, p. 13.

SELIGMANN, M., PREUD'HOMME, J-L. & BROUET, J-C. (1973) B and T Cell Markers in Human Proliferative Blood Diseases and Primary Immunodeficiencies, with Special Reference to Membrane Bound Immunoglobulins. *Transplantn Rev.*, **16**, 85.

SHELDON, P. J., PAPAMICHAIL, M., HEMSTED, E. H. & HOLBOROW, E. J. (1973) Thymic Origin of Atypical Lymphoid Cells in Infectious Mononucleosis. *Lancet*, i, 1153.

SHEVACH, E. M., JAFFE, E. S. & GREEN, I. (1973) Receptors for Complement and Immunoglobulin on Human and Animal Lymphoid Cells. *Transplantn Rev.*, **16**, 3.

SMITH, J. L., BARKER, C. R., CLEIN, G. P. & COLLINS, R. D. (1973) Characterisation of Malignant Mediastinal Lymphoid Neoplasm (Sternberg Sarcoma) as Thymic in Origin. *Lancet*, i, 74.

SPECIAL TECHNICAL REPORT (1974) Identification, Enumeration and Isolation of B and T Lymphocytes from Human Peripheral Blood. *Scand. J. Immunol.*, **3**, 521.

WILSON, J. D. & HURDLE, A. D. F. (1973) Surface Immunoglobulins on Lymphocytes in Chronic Lymphocytic Leukaemia and Lymphosarcoma. *Br. J. Haemat.*, **24**, 563.

YAMAMOTO, A. & KAWAMURA, A. (1970) Assessment of Conjugates. In *Standardisation in Immunofluorescence*. Ed. E. J. Holborow. Oxford: Blackwell Scientific Publications. p. 181.

YODOI, J., TAKATSUKI, K., AOKI, N. & MASUDA, T. (1974) Chronic Lymphocytic Leukaemia of T-cell Origin: Demonstration of Two Cases by the Use of Anti-thymocyte Membrane Antiserum. *Acta Haemat. jap.*, **37**, 46.

ZIEGLER, J., HANSEN, P. J. & PENNY, R. (1975) Intrinsic Lymphocyte Defect in Hodgkin's Disease: Analysis of the Phytohaemagglutinin Dose-response. *Clin. Immunol. & Immunopathol.* In the press.